# Faith, stigma, and resilience: a qualitative study of the experiences of mothers raising children with autism spectrum disorder in West Azerbaijan, Iran

**DOI:** 10.1186/s40359-026-05157-2

**Published:** 2026-07-09

**Authors:** Fatemeh Moghaddam-Tabrizi, Samira Orujlu, Fariba Hosseinzadegan, Mahdiyeh Abbasi, Mehran Soleymani

**Affiliations:** 1 Department of Consultation on Midwifery, School of Nursing and Midwifery, Urmia University of Medical Sciences, Urmia, Iran; 2 Reproductive Health Research Center, Clinical Research Institute, Urmia University of Medical Sciences, Urmia, Iran; 3 Department of Medical Surgical Nursing, School of Nursing and Midwifery, Urmia University of Medical Sciences, Urmia, Iran; 4 Department of Medical Surgical Nursing, School of Nursing and Midwifery, Tabriz University of Medical Sciences, Tabriz, Iran; 5 Department of Psychiatric Nursing, School of Nursing and Midwifery, Urmia University of Medical Sciences, Urmia, Iran; 6 Azarbaijan Shahid Madani University, Tabriz, Iran

**Keywords:** Autism spectrum disorder, Mothers, Caregiving, Stigma, Spirituality, Qualitative content analysis, Iran

## Abstract

**Background:**

Mothers of children with autism spectrum disorder (ASD) may experience substantial emotional, social, and practical challenges, compounded by societal stigma and cultural beliefs, especially in Iran. This study aimed to explore the experiences of Iranian mothers who have children with ASD, including the challenges they encounter, ways they cope, and how cultural and societal factors play a role in their experience.

**Methods:**

This qualitative descriptive study utilized purposive sampling to recruit 17 mothers of children with ASD from a single-center specialized treatment and rehabilitation setting in Urmia, Iran. Data were collected via individual, semi-structured interviews and subjected to qualitative content analysis.

**Results:**

Four main categories emerged from the qualitative analysis: (1) mothers’ perceptions of the causes of autism, (2) the evolving journey of coping with an autism diagnosis, (3) challenges faced by mothers of children with ASD, and (4) the profound and complex impact of autism on mothers’ lives. Regarding causal perceptions, mothers attributed autism to spiritual lenses, alternatively viewing it as a perceived divine purpose and meaning, framing autism as a test of faith, acceptance of preordained destiny, or perceived divine punishment and guilt. The coping trajectory advanced from initial confusion, shock, and protective concealment due to fear of stigma toward gradual acceptance and resource utilization. Key structural challenges included social stigma, limited public awareness, insufficient access to specialized centers, lack of psychological support, and significant financial strain. Finally, the condition profoundly disrupted maternal careers and family dynamics, leading to severe emotional distress, though mothers also reported finding existential meaning and personal growth through their caregiving role.

**Conclusions:**

This study underlines the significance of cultural and spiritual dimensions in shaping the experiences of Iranian mothers of children with ASD, highlighting both the challenges faced and sources of resilience identified. The findings indicate a need for increased societal awareness, culturally sensitive support, and improved access to services for mothers and families in comparable contexts.

**Supplementary Information:**

The online version contains supplementary material available at https://doi.org/10.1186/s40359-026-05157-2.

## Introduction

Autism Spectrum Disorder (ASD) is a lifelong neurodevelopmental condition characterized by persistent challenges in social communication, restricted interests, and repetitive behaviors [1]. Globally, the estimated prevalence of ASD stands at approximately 1 in 100 children [2]. In the United States, recent data from the Centers for Disease Control and Prevention (CDC) indicate a prevalence of 1 in 36 children aged 8 years [3]. Comparable trends are observed in the Middle East; in Iran, national registries and systematic reviews report a prevalence of about 1.3%, or 1 in 77 children [4]. Taken together, the rising prevalence of ASD worldwide highlights an urgent public health imperative: to address the long-term psychosocial impact on primary caregivers, who are central to daily care.

The impact of ASD profoundly disrupts the entire family network, affecting fathers, siblings, and extended kin; however, the daily operational and psychological burdens of caregiving fall disproportionately on mothers. In Iran, prevailing gender norms position mothers as the primary caregivers, while fathers are typically responsible for financial support. Regional studies indicate that mothers engage in substantially more daily care, resulting in a distinct caregiving role [5]. Although fathers and extended family influence the home environment, their direct involvement in clinical and behavioral management remains limited [5, 6]. This lack of familial participation often leads to maternal social isolation and increased exposure to external blame and affiliate stigma [6]. As a result, mothers are disproportionately vulnerable to chronic emotional distress and anxiety, underscoring the necessity of examining their unique experiences.

The well-being and family dynamics of mothers of children with ASD are substantially affected by social adversities, which are best understood through the conceptual lens of affiliate stigma -the process by which public prejudices are deeply internalized by primary caregivers. This stigma, frequently manifesting as societal judgment and misunderstanding of the child’s behavior, can lead to social isolation and feelings of rejection [7, 8]. Mothers may encounter negative perceptions from peers, relatives, and professionals, which convert public courtesy stigma into an internalized affiliate burden that impedes their ability to seek support and participate in community life [9, 10]. Furthermore, mothers of children with ASD face considerable systemic barriers to accessing adequate support, which intensifies the overall caregiving burden. Navigating complex healthcare and educational systems is often overwhelming, with many mothers reporting insufficient, inaccessible resources tailored to their needs [11]. As Luymes et al. [11] and Radev [12] highlight, these systemic inefficiencies are frequently regarded as the principal barrier, reinforcing the imperative for service delivery reform. Additionally, substantial financial burdens arising from therapies, medical care, and specialized interventions intensify hardship, often necessitating career compromises and exacerbating economic vulnerability [13–15]. Collectively, these factors underscore the urgent need for enhanced support structures and systemic reform to improve the well-being and family functioning of mothers raising children with ASD.

Despite the significant challenges they face, mothers of children with ASD often exhibit remarkable resilience and employ various coping mechanisms to navigate caregiving responsibilities within a non-linear family adaptation framework. Coping mechanisms such as faith, spirituality, and community-based support systems are often integral to maintaining emotional well-being and providing a sense of purpose amid caregiving responsibilities [16, 17]. Framing these efforts through cultural and spiritual meaning-making models suggests that some mothers rely on religious and spiritual practices to foster resilience and improve well-being [18, 19]. Social networks, including family members, healthcare professionals, and peer groups, are vital in reducing stress and offering emotional support [20, 21]. However, the extent to which these support systems can effectively alleviate distress is often dependent on prevailing cultural and social perceptions of disability and societal attitudes toward autism [15, 22].

To fully understand the experiences of mothers of children with ASD, it is important to consider both their coping strategies and the wider social and cultural context. While many studies in Iran and the region have described parenting stress, stigma, and religious coping [17, 23, 24], some important gaps remain. First, most research in the region tends to present Islamic spiritual and religious practices as universally positive and protective for families [17]. This overlooks the reality that for many mothers, faith can be both a source of comfort and a source of pain. The same religious beliefs that offer hope and strength can also lead to deep feelings of guilt, self-blame, and fears of divine punishment when caring for a child with ASD. Second, much of the research on Iranian ASD caregivers focuses solely on families living in large cities such as Tehran [5, 25]. This leaves out the voices of mothers in smaller cities and rural areas, where limited services, traditional gender roles, and unique local cultures create very different caregiving experiences.

This qualitative descriptive study directly addresses these empirical gaps by exploring the experiences of mothers of children with ASD in Urmia, a prominent border-proximate city in West Azerbaijan characterized by a unique multi-ethnic demographic context. By analyzing how these primary caregivers navigate geographically centralized healthcare shortages and structural isolation while reconciling conflicting spiritual attributions of disability, this study offers a nuanced conceptual model that extends beyond broad national generalizations. These findings can inform culturally synchronized, family-centered nursing protocols, localized mental health safety nets, and targeted community awareness campaigns in under-resourced provincial regions. Crucially, this study frames adaptation not as a static, linear endpoint achieved uniformly following diagnosis, but as a fluid, cyclical, and frequently incomplete process. By highlighting the overlapping realities of lingering social isolation, active concealment, and recurrent emotional crises alongside moments of profound meaning-making, the study captures the true non-linear complexity of the caregiving experience.

## Methods

### Study design

This study used a qualitative descriptive design to explore the experiences of mothers of children with ASD in Urmia, Iran. The approach was chosen to stay close to participants’ narratives and provide a low-inference summary of daily challenges and structural barriers [26]. To analyze the data, a conventional content analysis framework was systematically applied to interpret the data and summarize both manifest descriptions of physical or financial burdens and latent meanings of cultural and spiritual experiences [27]. Data collection took place between 2023 and 2024 at the Pishgamane Omid specialized center for the treatment, counseling, education, and rehabilitation of children with ASD. The study aimed to understand mothers’ perceptions, coping stages, challenges, and the impact of raising a child with ASD.

### Setting and participants

Purposive sampling was used to recruit participants from a single specialized provincial autism referral and rehabilitation center in Urmia, Iran. This centralized institutional setting predominantly serves an urban population, and all participating mothers were from urban families residing in this municipality. All participants were current members of the center whose children were receiving ongoing therapeutic, behavioral, or medical interventions there at the time of the study. Potential participants were approached face-to-face by the primary researcher in a private room at the facility during their routine clinical appointments. To ensure ethical transparency and address research positionality, one co-author served as the administrative manager of this specialized center, which facilitated institutional access to the registry.

Seventeen mothers of children diagnosed with ASD were selected to ensure diverse maternal and child characteristics and capture a broad spectrum of experiences. Characteristics considered for variation included maternal age, education level, working status, number of children, and child age, gender, and ASD severity (mild to severe). The mothers were all primary caregivers of a child with a confirmed ASD diagnosis. Data saturation was monitored throughout the inductive analysis process. Code and thematic saturation were achieved by the 15th interview, at which point no new conceptual categories emerged. To confirm this assessment, two additional validation interviews (16 and 17) were conducted, but they yielded no new insights. This judgment was tracked through a code recurrence matrix and team reflexivity logs. Demographic characteristics of the participants are detailed in Table 1. Mothers with a pre-existing, clinically documented history of major psychiatric illness or substance abuse before their child’s diagnosis were not eligible to participate. This exclusion criterion was evaluated during screening by cross-checking the mothers’ medical history records in the center’s institutional database and by an initial face-to-face health history intake interview. No formal diagnostic screening instruments were administered. It is critical to distinguish pre-existing clinical psychiatric history from the situational, severe emotional crises—including expressions of overwhelming distress, localized self-harm thoughts, and profound grief—captured during our data collection. These emergent psychological challenges represent acute situational trauma tied to the chronic caregiving burden, systemic isolation, and lack of support networks, rather than an underlying, pre-existing clinical psychiatric diagnosis.

**Table 1:** Demographic characteristics of participating mothers and their children with ASD

Characteristics	Mean ± SD *N* (%)
Mother	
Age, years	35.8 ± 5.2
Working status	
* Housewife*	9(52.9)
* Working*	8(47.1)
Education level	
* Illiterate*	1(5.9)
* Primary school*	3(17.7)
* High school*	4(23.5)
* University*	9(52.9)
Number of children	
* 1*	5(29.4)
* 2*	8(47.1)
* 3*	4(23.5)
Child	
Age, years	5.1 ± 1.7
Gender (male)	11(64.7)
Autism spectrum *	
* Mild*	4(23.5)
* Moderate*	6(35.3)
* Severe*	7(41.2)

*Childhood autism severity was extracted directly from the children’s medical records based on the official clinical diagnoses documented by a child psychiatrist

### Data translation and cross-language analysis

The primary data collection was linguistically multi-layered due to the bilingual demographic of the Urmia region. Based on participants’ explicit comfort and preference, interviews were conducted either entirely in Persian or in Azerbaijani Turkish. To establish a uniform and rigorous analytical workflow, those sessions conducted in Azerbaijani Turkish were first transcribed verbatim in the source language and then carefully translated into Persian by a bilingual research team member fluent in both regional languages. The qualitative content analysis was subsequently conducted on this unified Persian dataset. Finally, selected representative codes, subcategories, categories, and illustrative quotes were translated from Persian into English for manuscript presentation.

### Data collection

Data for this study were obtained through individual, semi-structured, in-depth interviews, which facilitated a rich and comprehensive exploration of the mothers’ experiences. Each interview lasted approximately 30 to 45 min. An initial interview guide was developed based on an extensive review of the existing literature and refined through preliminary discussions within the research team to ensure relevance and conceptual alignment. Throughout the seven-month data collection and analysis period, the research team held regular debriefing sessions to critique their engagement and promote reflexivity. This reflexive process supported the iterative adaptation of data collection strategies in response to emerging insights.

The semi-structured, open-ended questions were intentionally broad to empower participants to lead the narrative. Crucially, these guiding questions were followed by flexible, participant-driven probes adapted in real time in response to each mother’s immediate responses. These exploratory probes were strategically used to resolve potential conceptual overlaps and elicit deeper, more nuanced insights into complex personal domains. With participants’ written informed consent, all sessions were audio-recorded for accuracy, subsequently transcribed verbatim by the primary researcher, and verified for transcript fidelity. The comprehensive interview guide, along with exemplar probes, is presented in Table 2.

**Table 2:** Semi-structured interview guide and Exemplar Probes

Main Interview Questions	Exemplar Probes
1. Can you describe your experiences of living with a child diagnosed with autism?	-What did a typical day look like when you first noticed unusual behaviors?
2. How do you perceive and interpret your child’s autism?	-How do your internal, cultural, or spiritual beliefs shape this interpretation?
3. What were your initial feelings upon the autism diagnosis of your child?	-How did those feelings affect your decision to share or conceal the diagnosis?
4. How have you managed and coped with your child’s autism?	-What specific steps or programs led you toward gradual adaptation?
5. In what ways has autism influenced or affected your personal and social life?	-How do you navigate negative public judgments or family blame?
6. What challenges have you encountered while raising a child with ASD?	-How do regional resource scarcity and financial costs impact your family?
7. What are your expectations for overcoming the challenges you currently face with your child with ASD?	-What specific type of formal or financial support do you need most?
8. How has autism reshaped the meaning and purpose of your life?	-How has this journey impacted your career, marriage, and personal growth?

### Data analysis

Data analysis was facilitated using MAXQDA software solely as a systematic tool for data organization, retrieval, and code management; all interpretive analysis was explicitly executed by the research team following the qualitative conventional content analysis framework by Graneheim and Lundman [28]. To establish high analytical rigor, a collaborative, multi-stage coding process was employed. Initially, two researchers independently read the unified Persian transcripts multiple times to achieve immersion and performed line-by-line open coding to extract manifest and latent meaning units. Following independent coding, the two researchers held a series of collaborative meetings to cross-examine their preliminary codebooks and group descriptive codes iteratively based on semantic similarities. Disagreements regarding code definitions, boundary overlaps, or segment assignments were systematically resolved through reflexive dialogue and mutual consensus. Once code consistency was achieved, the wider research team was convened to review the evolving conceptual framework. Finally, the architecture of higher-order subcategories and main categories was collectively agreed upon by identifying cross-cutting patterns and checking category definitions against the raw text, ensuring that the final categories precisely reflected the participants’ authentic experiences.

### Trustworthiness

To establish methodological rigor and scientific integrity, trustworthiness was evaluated according to Lincoln and Guba’s criteria [29]: credibility, dependability, confirmability, and transferability .

#### Credibility

Credibility was ensured through sustained engagement with the research topic, participants, and data over a 7-month data-collection period. Investigator triangulation was achieved by involving multiple researchers in coding and analysis, along with regular cross-disciplinary meetings in which all team members discussed and refined the emerging categories. A formal member-checking process was conducted with four participating mothers. They were presented with their verbatim transcripts and a comprehensive Persian summary of the translated primary codes and subcategories. They confirmed that the generated categories captured their caregiving experiences and that no structural modifications or category realignments were required.

#### Dependability

Dependability was supported by detailed documentation throughout the data analysis process and transparent reporting. An explicit audit trail documented every phase of the research, including raw interview recordings, verbatim multi-language transcriptions, evolving iterations of the codebook in MAXQDA, and the meeting logs that tracked the thematic development of the final categories.

#### Confirmability

To ensure confirmability, team reflexivity sessions were continuously utilized during collaborative meetings to actively monitor and mitigate potential researcher positionality and preconceptions. This reflective process ensured that interpretation remained strictly rooted in the empirical evidence of the participants’ narratives rather than in the researcher’s subjective biases.

#### Transferability

While the qualitative descriptive nature of this study confines the data to a specific context, transferability was maximized by providing a rich, thick description of the unique regional and institutional caregiving environment in Urmia, Iran. Complete transparency regarding the purposive sampling selection criteria, socio-demographic variations (Table 1), and traditional structural constraints was provided to allow future investigators to assess the applicability of these findings to similar provincial or under-resourced settings.

### Ethical considerations

Ethical approval for this study was obtained from the Ethics Committee of the Urmia University of Medical Sciences. All participants provided written informed consent prior to data collection, and confidentiality was maintained by utilizing numerical codes instead of identifying information. The written informed consent obtained from all participants explicitly included permission for the use and dissemination of their anonymized direct quotations in scientific publications. To guarantee participant anonymity within this single-center setting, all direct quotations are presented using standardized alphanumeric labels (e.g., M1, M2) and broad age ranges for both mothers and children (e.g., M1, maternal age group: 30–35, child aged 3–5) instead of names or exact ages. Because of the sensitive nature of caregiving burdens, a formal distress protocol was established. If a participant experienced severe distress or disclosed self-harm/homicidal ideation, the interview was immediately paused to provide psychological first aid and an immediate crisis referral to the center’s on-site clinical psychologist for formal risk assessment, safety planning, and structural follow-up.

## Results

Seventeen mothers of children with ASD (mean age: 35.8 years) participated in this study. The qualitative analysis revealed four main categories: mothers’ perceptions of the causes of autism, the evolving journey of coping with an autism diagnosis, challenges faced by mothers of children with ASD, and the profound and complex impact of autism on mothers’ lives (Table 3).

**Table 3:** Main categories, subcategories, and representative qualitative quotations

Main Categories	Subcategories	Representative Qualitative Quotations
1. Perceptions of Autism Causes	1.1. Perceived Divine Purpose & Meaning	“God is trying to fix something with this. He is trying to make something purposeful. I pray and trust in Him. This is all God's wisdom.”
	1.2. Framing Autism as a Test of Faith	“I came to understand that this child represents a personal test for me, and I must strive to approach and overcome this test successfully.”
	1.3. Acceptance of Preordained Destiny	“This is my destiny and fate; it feels as though God has ordained it this way.”
	1.4. Perceived Divine Punishment & Guilt	“I curse God for why I have to be like this... Maybe God is punishing me.” / “God wants to punish me in this way so that my sins will be reduced...”
2. Evolving Journey of Coping	2.1. Initial Confusion and Doubt	“I myself had suspected my child's unusual behavior. But I told myself, he'll grow up, he'll get better... I didn't think about it at all.”
	2.2. Impact of Diagnosis: Shock & Denial	“I didn't want to accept this. I kept telling myself that it's impossible for my child to have autism. Is it possible?”
	2.3. Concealing the Diagnosis: Fear of Stigma	“I didn’t tell anyone at home—not even my mom... I kept it to myself because I didn’t want others to change their thoughts or opinions...”
	2.4. Gradual Acceptance and Adaptation	“It was very difficult to accept, but over time, we came to terms with it... I realized that I needed to approach him differently.”
	2.5. Seeking Support and Resources	“The Tehran Autism Association helped us immensely... At one point, our hope was at zero. With their advice, they raised our hope to 50.”
3. Compounding Caregiving Challenges	3.1. Social Stigma and Misconceptions	“My husband’s family said... ‘the issue must have come from your side... It’s your fault.’” / “My sister... says, ‘I know he’s mentally retarded.”
	3.2. Lack of Public Awareness & Acceptance	“Society doesn’t accept it at all... when I’m riding a bus... my son may make noise or cry, and everyone looks at me.”
	3.3. Insufficient Access to Centers	“The number of educational centers must be increased. Some families live far from these centers and can’t afford to travel.”
	3.4. Inadequate Psychological Support	“We mothers of autistic children need support. We need to feel empowered and given hope. We also need advice to maintain motivation...”
	3.5. Financial Strain	“The financial strain is overwhelming. We’ve cut all our other living expenses to focus on our child. Each therapy session is incredibly expensive.”
4. Profound Impact on Mothers' Lives	4.1. Social Isolation & Role Disruption	“I have a master’s degree in chemistry. I used to teach at a university. Now, I don’t go to work anymore because of my son.”
	4.2. Psychological & Emotional Challenges	“I’m very aggressive. I hit myself... I have suicidal thoughts every day. Many times, I’ve even thought about killing my child and myself.”
	4.3. Impact on Family Dynamics	“Our family was very unstable. There was a lot of tension between me and my husband until our case ended up in court and the police station.”
	4.4. Finding Meaning and Purpose	“My life becomes meaningful when I see my daughter succeed... Having an autistic child is a new experience that has allowed me to achieve personal growth.”

### Mothers’ perceptions of the causes of autism

Mothers’ perceptions of the causes of autism were often shaped by their faith and spiritual beliefs. For some, their child’s diagnosis was seen as part of a greater divine purpose, offering comfort and meaning to their journey. Others interpreted it as a test of faith, requiring strength and perseverance. Some mothers accepted autism as part of their destiny, believing it was preordained by a higher power. However, others struggled with feelings of divine punishment, experiencing guilt or resentment over their circumstances. The following sections explore these interpretations in more detail, providing insight into the emotional and spiritual responses of these mothers.

#### Perceived divine purpose and meaning

For some mothers, autism was perceived as divinely ordained, offering a sense of comfort and meaning amidst the challenges. These mothers sought solace in their faith, trusting in a higher purpose. As one mother explained, “God is trying to fix something with this. He is trying to make something purposeful. I pray and trust in Him. This is all God’s wisdom.” (M1, maternal age group: 30–35, child aged 4–6). This perspective reflects a belief that autism is not a random occurrence but rather an integral part of a larger, divine design. This quotation demonstrates how establishing a divine purpose serves as an initial psychological defense, transforming chronic sorrow into a spiritually sanctioned mission.

#### Framing autism as a test of faith

Several mothers viewed raising a child with autism as a profound test of faith, requiring significant effort and resilience to manage effectively and achieve positive outcomes. One mother reflected, “I came to understand that this child represents a personal test for me, and I must strive to approach and overcome this test successfully.” (M4, maternal age group: 25–30, child aged 5–7). This reflection illustrates a cognitive shift from merely seeking “divine comfort” to actively embracing a spiritual obligation, transforming the caregiving burden into a trial of personal resilience.

#### Acceptance of preordained destiny

A sense of acceptance and surrender was evident in mothers who perceived autism as an integral part of their destiny. This perspective reflects a deep faith in a higher power and a belief that all aspects of life are preordained. As one mother articulated, “This is my destiny and fate; it feels as though God has ordained it this way.” (M7, maternal age group: 35–40, child aged 7–9). This suggests a belief in a divinely guided life path, where autism is not an unexpected deviation but a predetermined part of their journey. This narrative marks a transition from actively “overcoming a test” to psychological surrender, where accepting a predetermined fate reduces cognitive resistance to the diagnosis.

#### Perceived divine punishment and guilt

In contrast to the perspectives of acceptance and purpose, some mothers interpreted their child’s autism as a form of divine punishment, reflecting feelings of injustice and prompting them to question their relationship with God. One mother remarked, “I curse God for why I have to be like this. I don’t know. Maybe God is punishing me.” (M2, maternal age group: 30–35, child aged 3–5). Another mother said, “God wants to punish me in this way so that my sins will be reduced a little.” (M9, maternal age group: 40–45, child aged 6–8). These narratives highlight the dark side of spiritual causation, where interpreting ASD through a punitive lens exacerbates internalized guilt, leading to acute emotional crises and theological struggle rather than adaptation.

### The evolving journey of coping with an autism diagnosis

Mothers navigating their child’s ASD diagnosis experienced complex, non-linear emotional phases. Initially, some primary caregivers reported uncertainty, frequently rationalizing early developmental delays or behavioral flags as temporary milestones. When the diagnosis was formally established, this clinical confirmation frequently precipitated acute shock and denial, leading to emotional turmoil and difficulty accepting the reality. Some mothers chose to conceal the diagnosis out of fear of social stigma and judgment. Over time, gradual acceptance set in as they learned to adapt to their child’s needs and found new ways to connect. To support their child’s development, some sought educational resources and counseling, gaining valuable guidance and hope through specialized programs. These subcategories mapped a sequential trajectory of psychosocial transition; the cognitive defense of early normalization collapsed into acute emotional crisis upon diagnosis, which initially triggered social withdrawal before evolving into constructive behavioral adaptation and external resource mobilization.

#### Initial confusion and doubt

Before a formal diagnosis, some mothers reported experiencing confusion and doubt regarding early signs of their child’s atypical behavior. As one mother explained, “I myself had suspected my child’s unusual behavior. But I told myself, he’ll grow up, he’ll get better. I had heard the word autism, but I didn’t think my child had it. There were no children like that around us. We didn’t have any family marriages. I didn’t think about it at all.” (M3, maternal age group: 25–30, child aged 4–6). This suggested a tendency to normalize early differences, particularly in the absence of readily available information or support. This narrative illustrated how a lack of localized awareness drove protective rationalization, delaying early screening by attributing symptoms to temporary developmental phases.

#### The impact of diagnosis: shock and denial

The formal diagnosis often triggered profound shock and denial, representing a significant emotional turning point. Mothers described struggling to accept the reality of the diagnosis. One mother shared, “I didn’t want to accept this. I kept telling myself that it’s impossible for my child to have autism. Is it possible?” (M5, maternal age group: 35–40, child aged 3–5). This reaction highlighted the initial disbelief and emotional turmoil that can accompany an ASD diagnosis. This quote captured the cognitive fragmentation that occurred when formal clinical reality shattered the mother’s previous strategies of normalization.

#### Concealing the diagnosis: fear of stigma

Some mothers described their inclination to conceal their child’s autism diagnosis due to fears of social stigma, judgment, and negative changes in how others might perceive their child. One mother recounted, “I didn’t tell anyone at home—not even my mom—when I took my son to the doctor. My mom was sitting outside the office and asked what the doctor had said, but I didn’t tell her. No one in the family knows. I kept it to myself because I didn’t want others to change their thoughts or opinions about my child.” (M8, maternal age group: 30–35, child aged 5–7). This revealed the lengths to which some mothers went to protect their child from potential negative reactions. This profound level of secrecy highlighted how anticipated family and social stigma acted as an immediate barrier, forcing mothers into operational isolation to shield their child.

#### Gradual acceptance and adaptation

Over time, some mothers described a gradual process of acceptance and adaptation to their child’s condition. This often involved learning to understand their child’s unique needs and developing new ways to connect and communicate. As one mother stated, “It was very difficult to accept, but over time, we came to terms with it. We eventually accepted it as a condition my son had.” (M6, maternal age group: 35–40, child aged 6–8). Another mother described her journey of understanding her child’s behavior: “Initially, I struggled to communicate with my son. I didn’t understand him, and he didn’t understand me. But gradually, I accepted his condition and realized that I needed to approach him differently.” (M11, maternal age group: 25–30, child aged 4–6). These accounts illustrated the emotional work involved in accepting the diagnosis and adapting to the realities of raising a child with ASD. This transition demonstrated a shift from passive grief to active behavioral reframing, in which understanding the child’s unique communication patterns fostered relational resilience.

#### Seeking support and resources

Seeking educational resources and support systems emerged as a crucial component of coping and adaptation. Mothers emphasized the importance of specialized programs, counseling, and peer support in navigating the challenges of ASD. One mother highlighted the significance of such support: “The Tehran Autism Association helped us immensely and provided guidance. At one point, our hope for the future was at zero. With their advice, they raised our hope to 50.” (M10, maternal age group: 40–45, child aged 7–9). Others emphasized the importance of counseling: “I sought advice from multiple counseling centers. I was in a state of turmoil and agitation, trying to find a way to help my child and get him out of this situation.” (M12, maternal age group: 30–35, child aged 5–7). These experiences emphasized the vital role of support systems in empowering mothers and fostering resilience. This final phase highlighted that true psychological adaptation is rarely achieved in isolation; accessing external institutional support effectively converted internal resilience into measurable action and renewed hope.

### Challenges faced by mothers of children with ASD

Mothers of children with ASD faced a range of significant challenges. Social stigma and misconceptions often led to blame, judgment, and hurtful labels from family and society. These difficulties were worsened by a widespread lack of awareness about autism, which resulted in negative reactions to their child’s behavior in public settings. Limited access to specialized educational centers, especially in rural areas, further complicated their ability to support their child’s needs. Additionally, some mothers lacked adequate psychological and informational support, making it difficult to maintain hope and resilience. Financial strain was a significant systemic burden, as the compounding costs of private behavioral rehabilitation and speech therapy placed immense pressure on households, compelling families to divert economic resources from baseline living expenses. These challenges operate as compounding systemic barriers; interpersonal stigma within the family scaled up to public rejection in society, both of which were severely exacerbated by geographical isolation from care centers and a lack of professional guidance, ultimately culminating in a state of chronic financial exhaustion.

#### Social stigma and misconceptions

Social stigma and misconceptions surrounding autism created a pervasive burden for mothers. Judgmental attitudes, often accompanied by blame and hurtful labels, were common within families and the broader community. One mother recounted, “My husband’s family said, “We don’t have any problems; the issue must have come from your side and been passed on genetically to the child. It’s your fault.” (M2, maternal age group: 30–35, child aged 3–5). Another mother shared her experience, stating, “My sister, when she sees my son, says, ‘I know he’s mentally retarded. It’s obvious from his appearance.” (M14, maternal age group: 35–40, child aged 6–8). These experiences illustrated the damaging impact of social stigma and the lack of understanding surrounding autism. Here, social stigma was conceptualized strictly as an external, structural societal challenge and negative public judgment imposed dynamically on the mothers. These painful familial experiences illustrated that stigma was most damaging when it originated from close kin, weaponizing genetic blame to strip mothers of their immediate emotional safety net.

#### Lack of public awareness and acceptance

The lack of public awareness and acceptance of autism further exacerbated the challenges faced by mothers. Negative reactions to their child’s behavior in public settings, often stemming from misunderstanding, could lead to feelings of isolation and shame. One mother explained, “Society doesn’t accept it at all. For example, when I’m riding a bus or attending a social gathering, my son may make noise or cry, and everyone looks at me. Society simply doesn’t accept this.” (M15, maternal age group: 25–30, child aged 4–6). This highlighted the need for increased public education and awareness campaigns to foster greater understanding and acceptance of individuals with ASD. This quotation emphasized how public environments became spaces of hyper-surveillance and silent exclusion, turning ordinary daily transits into sources of profound psychological distress.

#### Insufficient access to specialized educational centers

Limited access to specialized educational centers, particularly in rural areas, presented a significant barrier to mothers seeking appropriate support for their children. The scarcity of such centers, coupled with geographical distance and financial constraints, restricted access to crucial resources. One mother suggested, “The number of educational centers must be increased. Some families live far from these centers and can’t afford to travel. While some endure these hardships to access services, others aren’t even aware of the existence of such centers.” (M13, maternal age group: 40–45, child aged 8–10). This signified the need for increased investment in specialized services and improved access for families, regardless of location or socioeconomic status. This insight highlighted structural and geographical inequities, where institutional centralization shifted the logistical and physical burden entirely onto marginalized households.

#### Inadequate psychological and informational support

Mothers also expressed a need for greater psychological and informational support to help them navigate the challenges of raising a child with ASD. Some mothers reported feeling overwhelmed and lacking the resources to effectively advocate for their child’s needs. One mother noted, “We mothers of children with ASD need support. We need to feel empowered and given hope. We also need advice to maintain motivation because some mothers quickly become discouraged by the demands of treatment.” (M16, maternal age group: 30–35, child aged 5–7). This highlighted the importance of providing comprehensive support services, including counseling, education, and peer support groups, to empower mothers and enhance their well-being. This reflection indicated that without targeted motivational and educational interventions, the chronic and exhausting nature of ASD therapies risked causing early maternal burnout.

#### Financial strain

The interviews underscored the significant financial burden of raising a child with ASD, with the high costs of educational and therapeutic sessions often forcing families to reduce spending in other areas of their lives. One mother described her family’s struggles, stating, “The financial strain is overwhelming. We’ve cut all our other living expenses to focus on our child. Each therapy session is incredibly expensive. I wish there were a source of financial support for families like ours.” (M8, maternal age group: 30–35, child aged 5–7). This highlighted the need for financial assistance programs and affordable access to essential services for families raising children with ASD. This quotation grounded the ultimate systemic trap; the compounding costs of private rehabilitation create severe household deprivation, legally and economically limiting the family’s socioeconomic survival.

### The profound and complex impact of autism on mothers’ lives

The impact of autism on various aspects of a mother’s life was profound and far-reaching. Some mothers experienced social isolation and disruption of their personal and professional roles, often withdrawing from social interactions or giving up career aspirations to care for their child. The psychological and emotional toll was significant, leading to anger, grief, depression, and even self-harm or suicidal thoughts, underscoring the need for mental health support. Family dynamics were also strained, with marital tensions, neglect of other siblings, and anxiety about the child’s future becoming common challenges. Despite these difficulties, some mothers found meaning and purpose through their child’s progress, experiencing personal growth, spiritual development, and fulfillment in their caregiving role. The following sections explore these impacts in more detail, highlighting the complex ways autism reshaped mothers’ lives and the resilience they displayed. Crucially, these subcategories captured the ultimate, cascading impact of chronic caregiving; the forced sacrifice of maternal identity and professional roles precipitated severe internal psychological trauma and existential crises, which inevitably spilled over into outer relational systems, causing severe disruptions in family and marital dynamics. However, this destructive cycle was bounded by a capacity for post-traumatic growth, where incremental child progress enabled ultimate spiritual and personal transcendence.

#### Social isolation and role disruption

The findings revealed that mothers often experienced withdrawal from social interactions and a disruption of their educational and career aspirations due to the demands of raising a child with ASD. The diagnosis also significantly affected their relationships with others. One mother shared, “I have a master’s degree in chemistry. I used to teach at a university. Now, I don’t go to work anymore because of my son.” (M17, maternal age group: 35–40, child aged 6–8). Another mother reflected, “After my son’s diagnosis, I became a social outcast. I don’t attend parties anymore because people look at me negatively.” (M5, maternal age group: 35–40, child aged 3–5). These experiences illustrated the significant sacrifices mothers often made and the social isolation that could result from the demands of caregiving and societal perceptions. Here, social isolation is explicitly operationalized as a personal and relational consequence, directly reflecting the profound long-term impact of the caregiving environment on the mother’s personal life. These powerful narratives highlight the erosion of individual identity, where mothers must entirely forfeit their professional achievements and undergo forced social erasure to sustain their caregiving responsibilities.

#### Psychological and emotional challenges for mothers

Mothers of children with ASD faced intense psychological and emotional challenges, including anger, grief, and emotional exhaustion as they adjusted to their child’s condition. Some mothers described profound struggles, such as self-harm, a crisis of faith, and even suicidal thoughts, highlighting the significant emotional toll of their circumstances. Additionally, reduced self-esteem and persistent fatigue stemmed from the constant demands of caregiving, societal judgment, and personal sacrifices. One mother expressed, “I’m very aggressive. I hit myself. I throw things. Autism is very difficult for me. I was severely depressed for a while.” (M2, maternal age group: 30–35, child aged 3–5). Another mother disclosed, “I have suicidal thoughts every day. Many times, I’ve even thought about killing my child and myself.” (M12, maternal age group: 30–35, child aged 5–7). These deeply personal accounts underscored the critical need for mental health support for mothers of children with ASD. These devastating expressions of self-harm and filicide-suicide ideation underscored that role erasure and chronic isolation eventually turned inward, manifesting as severe psychiatric trauma that threatened the survival of both mother and child.

#### Impact on family dynamics

Raising a child with ASD could significantly strain family dynamics. Marital tensions, conflicts between parents, and the potential neglect of other siblings were common challenges. Families often experienced a shift in priorities, with the needs of the child with ASD becoming the central focus. One mother shared, “Our family was very unstable. There was a lot of tension between my husband and me until our case ended up in court and the police station.” (M8, maternal age group: 30–35, child aged 5–7). Another mother reflected, “I had forgotten about my daughter. All my focus was on my son.” (M6, maternal age group: 35–40, child aged 6–8). Also, a mother noted, “All I could think about was my son moving forward and progressing. My son became the first and main priority in my life.” (M4, maternal age group: 25–30, child aged 5–7). These experiences illustrated the profound impact on family relationships and the need for support systems that address the needs of all family members. This quotation mapped how internal emotional trauma destabilized external relationships, creating systematic family fragmentation that manifested as legal-marital dissolution and the inadvertent emotional neglect of siblings.

#### Finding meaning and purpose

The subcategory of meaning and purpose in life demonstrated how mothers of children with ASD found meaning through their child’s progress, often deriving motivation and a sense of fulfillment from their development. This process emphasized love, spiritual growth, and personal transcendence, as mothers strived for personal growth through caregiving. Some mothers described this experience as a journey of learning and self-discovery, which enriched their lives despite the associated challenges. One mother stated, “My life becomes meaningful when I see my daughter succeed. For a while, I wished my daughter would talk. When I get a positive reaction from her, my life moves forward.” (M7, maternal age group: 35–40, child aged 7–9). Another mother reflected, “Having a child with ASD is a new experience that has enriched my life and allowed me to achieve personal growth.” (M11, maternal age group: 25–30, child aged 4–6). These accounts highlighted the capacity for resilience and the ability to find meaning and fulfillment even amidst adversity. This final phase provided a counter-narrative of post-traumatic growth, demonstrating that when systemic challenges were managed, minor developmental milestones could reconstruct maternal meaning, turning chronic sorrow into personal transformation.

## Discussion

This study explored the context-specific experiences of Iranian mothers of children with ASD at a specialized center in Urmia, Iran. Conscious of the non-linear nature of caregiver coping, the narratives show that adaptation is neither uniform nor complete; instead, these mothers cycle through phases of cognitive acceptance, protective concealment, and localized resilience while facing chronic structural barriers and systemic stigma. The findings also reveal a cultural duality in how mothers interpret autism, spanning perceived divine purpose, a test of faith, preordained destiny, perceived divine punishment, and guilt. Their emotional journey moved from initial confusion and denial to intentional concealment due to intense social stigma, and eventually toward localized acceptance. While caregiving brought severe psychological strain, social isolation, and disrupted family dynamics, it also revealed a capacity for meaning-making. Rather than offering broadly representative claims for all Iranian mothers, these localized insights underscore the transferable value of understanding interactional stigma and the need for targeted, family-centered support systems within this specific cultural context.

This study highlights the complex duality of spirituality, demonstrating that mothers’ faith does not merely serve as a passive coping mechanism but also functions as both a source of resilience and distress. While framing autism as a perceived divine purpose or a test of faith provided solace and emotional stability—consistent with global literature on faith-driven resilience [16, 30]— interpreting the diagnosis as a perceived divine punishment elicited profound guilt and spiritual resentment. This challenging aspect aligns with findings from culturally conservative contexts, where societal blame often reshapes religious interpretations [17, 31]. Rather than treating these beliefs as static, our findings show that mothers dynamically reframed punishment into existential tests, fostering post-traumatic growth. Consequently, these insights offer context-sensitive directions for designing faith-informed, family-centered support programs that address both the comforting and distressing dimensions of maternal spirituality.

The emotional trajectory of the participating mothers evolved nonlinearly from immediate shock and denial toward protective concealment and, eventually, localized adaptation. In this cultural context, initial psychological distress was intensified by anticipated social stigma, leading mothers to use concealment of the diagnosis as a primary defense against societal judgment. This self-imposed isolation and its detrimental effects on maternal well-being echo findings in similar culturally conservative settings [32, 33], where intense stigma heavily impedes the early adaptation process [34]. Crucially, the transition from concealment to acceptance was facilitated by psychological reframing and spiritual reliance, strengthening parent-child bonds and fostering personal growth—aligning with established caregiving transition frameworks [35, 36]. Consequently, there is a need for accessible, community-based support systems and peer support networks tailored to the region’s sociocultural dynamics.

The participating mothers navigated a compounding triad of challenges: intense social stigma, limited public awareness, and severe structural-financial strains. In this context, public misconceptions often translated into direct judgment and blame from both extended family and the wider community, forcing mothers into isolation. This localized experience of interactional stigma echoes findings from other culturally conservative settings where public misunderstanding diminishes maternal well-being [31, 33, 37]. Furthermore, the limited availability of specialized educational and therapeutic centers in the region—compounded by the logistical and financial burdens of commuting from rural areas—escalated parental stress. These structural and financial hardships mirror the caregiving strains documented globally [38–40]. Consequently, these findings highlight that addressing maternal burden in this region requires a dual approach: expanding access to geographically accessible, affordable specialized services and implementing targeted, context-sensitive public awareness initiatives to mitigate societal stigma.

The caregiving journey imposed a profound psychological toll on the participating mothers, manifested through chronic emotional exhaustion, isolation, and clinically significant distress. Crucially, the sub-themes revealing severe distress, including self-harm or overwhelming psychological crises, represent critical clinical indicators rather than isolated events. In this context, these severe emotional reactions are exacerbated by constant societal scrutiny and deep role disruption, echoing documented links between intense caregiver burden and compromised maternal mental health [23, 41]. These clinically significant findings underscore an urgent, pragmatic need to integrate routine mental health screening and structured psychiatric referral pathways directly into pediatric autism care settings. Addressing this acute distress requires accessible, family-centered psychological support, such as counseling and crisis-intervention programs, to mitigate the psychological toll before it escalates into severe clinical conditions.

Beyond individual psychological strain, raising a child with ASD altered family dynamics, introducing marital tension and the perceived neglect of typically developing siblings. This instability and heightened parental conflict align with established literature on the cascading effects of developmental conditions on family systems [42, 43]. However, alongside these vulnerabilities, the narratives revealed a striking capacity for post-traumatic growth; mothers reframed their roles to derive existential meaning, pride, and spiritual purpose from their caregiving journey [44, 45]. Consequently, these interwoven findings of vulnerability and resilience advocate for a shift toward family-centered support models. Such initiatives should address marital and sibling dynamics and leverage mothers’ spiritual and psychological resilience to foster sustainable family well-being.

## Limitations

Several limitations must be acknowledged in interpreting the findings of this study. First, the sample was recruited from a single specialized center in Urmia, West Azerbaijan province, had a relatively small sample size (*N* = 17), and focused exclusively on maternal perspectives. Given Iran’s extensive ethnic, linguistic, and religious diversity, this predominantly Azeri-Turkish, localized context limits the direct transferability of the findings to mothers from other regions (e.g., Persian or Sunni populations) or to those disconnected from formal services. Second, this study is subject to potential self-selection and social desirability biases, as participating mothers might hold different viewpoints or cross-cultural adaptive experiences compared to those who declined. Third, linguistic challenges arose from translating narratives across Turkish, Persian, and English, which may have flattened specific semantic meanings during analysis. Finally, the strict exclusion of mothers with pre-existing psychiatric illness represents an operational limitation, meaning our data captures severe situational distress but underrepresents caregivers with clinical-grade, formally diagnosed mental health conditions. Future qualitative inquiries should adopt multi-regional and multi-center designs to capture broader sociocultural and sub-cultural variations across Iran.

## Conclusions

This study explored the lived experiences of Iranian mothers of children with ASD, highlighting how cultural and spiritual beliefs shaped their perceptions and coping strategies. While faith often provided resilience and meaning, it could also evoke guilt or a sense of divine punishment. Social stigma and limited awareness of autism intensified the emotional and psychological challenges these mothers faced, with many choosing to conceal their child’s condition to avoid judgment and social ostracization. Financial strain, coupled with limited access to specialized educational resources, particularly in rural areas, created additional hardships for these families. Despite these challenges, the mothers in our study demonstrated resilience and strength, navigating the transition from initial confusion and denial to eventual acceptance and personal growth. This study underscores the need for culturally sensitive interventions, including targeted educational programs, accessible peer support groups, and readily available mental health resources, to alleviate the emotional, social, and financial burdens these families experience. Comprehensive support systems that address both the practical and emotional needs of mothers of children with ASD are essential for improving their overall well-being. The findings of this study call for increased societal awareness and enhanced institutional support for families of children with autism.

## Supplementary Information

Supplementary Material 1.

## Data Availability

Data are available upon request from the corresponding author.

## Abbreviation

ASD: Autism Spectrum Disorder
