# Supplementary material for: Faith, stigma, and resilience: a qualitative study of the experiences of mothers raising children with autism spectrum disorder in West Azerbaijan, Iran
Source: BMC Psychol. 2026 Jul 9;14:1423. doi: 10.1186/s40359-026-05157-2 (PMC13632098; doi:10.1186/s40359-026-05157-2)
Supplement: Supplementary file 1 [file 40359_2026_5157_MOESM1_ESM.docx]

When my son was 18 months old, he was diagnosed with autism. It felt like we had fallen into a swamp. Accepting it was incredibly difficult. As a mother, I accepted it sooner, but his father refused to accept it at all. It created a lot of tension in our lives. For a while, we constantly argued and fought. Eventually, he accepted it too, and our lives changed drastically. It took a long time to return to a normal routine, but that tension still exists.

What were those tensions?
The way people looked at us, their hurtful comments. They blamed me, saying I was the reason my child turned out this way. Those remarks led to constant arguments at home. My child couldn’t sit up or crawl on his own, yet people blamed me for it, claiming I hadn’t let him do those things. I kept saying my child had a problem and needed a doctor, but they dismissed it, accusing me of trying to label my child as sick. Eventually, I insisted, and we took him to the doctor, where his condition was confirmed. My husband's family claimed that since they didn’t have any issues, the problem must have come from my side. They said it was my fault. But now, they have accepted it.

I had an emergency cesarean section because the amniotic fluid had dried up. They terminated my pregnancy at 36 weeks.

How did this affect different aspects of your life?
My life was much better in all aspects before — socially, emotionally, even in my relationship with my husband. After the diagnosis, I avoided social gatherings for a while because people stared at me and asked questions about my son’s condition, what the doctor had said, and whether he would improve. I couldn’t handle those questions. In the beginning, I compared my son to other children and felt extremely sad. Now I no longer compare. I just accept that my child is who he is.

I can’t change how people look at me, but I’ve changed myself. I don’t pay attention to them anymore. I treat my child as if he’s just a normal kid. I shower him with love and praise so much that no one dares to say he has autism. I’ve acted this way for so long that now no one even asks about my son’s condition. Just yesterday, I went to a family gathering for the first time in a long while. The other children wouldn’t go near my son, saying they were afraid of him. At first, I used to cry when I came home from situations like that. But now I tell myself they’re just kids — they don’t understand.

What made you so resilient? Who helped you?
I helped myself.

What about your marital relationship?
Things were bad at first, but it’s better now, although not quite like it was before our son was born. My son is now two years and eight months old.

I’m a homemaker. I had found a job and wanted to start working, but after my son’s diagnosis, I couldn’t pursue it. I have a degree in architecture, but I couldn’t continue my studies because of my son.

For a long time, I struggled with severe depression and gained a lot of weight. But now I’ve started dieting and going to the gym. I’ve changed myself. I did it all alone.

Did you seek counseling or take medication for your depression?
No.

How are you now?
I’m fine.

I used to get angry often. I would hit myself, throw things, and break objects. Autism felt overwhelming for me. In the beginning, I even cursed God, questioning why this happened to me. My faith was shaken.

My husband, his family, and people’s words contributed to my severe depression. My husband would accept our son’s condition one day and deny it the next. He would lash out at me, blaming me for our son’s condition, then later regret it. His role wasn’t positive at first, but now he’s better.

When my son was first diagnosed, I didn’t tell anyone. I was alone. No one around me even knew what autism was. The day the doctor confirmed his diagnosis was the hardest day of my life. I walked home alone, carrying my child in my arms, crying the whole way. When I got home, I didn’t tell anyone. I just cried and lashed out. My mother eventually found out, and she’s been incredibly supportive. She watches my son so I can sleep or take care of other things.

My husband keeps his sadness to himself. He doesn’t express it openly, but I know he feels regret. Our son doesn’t say “Mama” or “Papa,” which makes my husband upset. But when my husband comes home from work, my son lights up with joy.

When we would go out in public, at first I felt ashamed. But now, even if people find out, it doesn't bother me anymore. Well, eventually he'll get better, and if he doesn't, that's just the way it is. What can I do? I'm not going to abandon my child for the sake of others. Now I've become much calmer and more relaxed, and I give my son a lot of love.

I’m hopeful he will improve. My son is tall, and when I say in public that my son will become a volleyball player, some people mockingly say, "Inshallah." I can tell from their faces what they mean. But I know that one day my son will be a volleyball player. In family gatherings, I’ve often been blamed, with people saying it’s my fault my son is ill, and sometimes I’ve secretly wished worse things would happen to them. The sympathetic looks from others, when they say "God willing, he’ll be healed," hurt me more than a hundred insults.

Early on, I avoided social gatherings. My mother told me, "How long can you keep this up? You need to accept that your child is just the way he is." I’m still bothered by people’s questions—whether my child will get better or not, and other intrusive remarks. Now, half of the relatives don't even know about his autism, or they whisper about it among themselves.

I'm very hopeful for my son. At first, I would respond harshly to people's behavior. Now I’m indifferent. I've accepted it. Others haven't reached that understanding yet. Why do people keep adding salt to my wounds?
